# Psychological impact of the covid 19 pandemic on health care workers

**DOI:** 10.1192/j.eurpsy.2023.982

**Published:** 2023-07-19

**Authors:** W. Ayed, S. Chebbi, A. Ayadi, S. Ayari, K. Hazem, I. Magroun

**Affiliations:** Occupational health departement, University of Tunis El Manar - faculty of Medicine of Tunis, Ariana, Tunisia

## Abstract

**Introduction:**

The COVID 19 pandemic had a significant psychological impact worldwide. Health care workers (HCWs) were the most affected because of the pandemic burden and occupational exigencies.

**Objectives:**

To describe epidemiological characteristics of HCWs with post COVID19 anxiodepressive disorders.

**Methods:**

A descriptive cross-sectional study was carried out. It included HCWs of a university hospital who consulted the Occupational Medicine Clinics for the three-month post-COVID‘s medical visit. The study was carried out during the period March 2020 to January 2022. The data was collected using a questionnaire including socio-occupational and medical characteristics. Psychometric evaluation was carried out using « the Hospital Anxiety and Depression Scale »

**Results:**

We have collected 164 HCWs. The sex ratio (M/F) was 0.29. The average age was 41±9.8 years. They belonged to the pneumology (27%), intensive care (11%) and biology laboratory (11%). The prevalence of anxiety and depression was 34% and 30% respectively. We found an association between sleep disorders and anxiety (p=0.000), OR=5 IC95%[2.4-10.3] and depression (p=0.000), OR= 4 IC 95%[2.0-9.3]. We found an association between anxiety and persistent fatigue (p=0,000), OR=4[2,0-8,6], anxiety and concentration and memory difficulties (p=0,000), OR=3 IC 95%[1,7-6,9]. Referral to psychiatric consultations were done in 16% of the cases.

**Conclusions:**

Post-COVID anxiety disorders were frequent among HCWs and associated with neurocognitive disorders. Psychiatric support and early treatment are necessary to prevent mental deterioration.

**Disclosure of Interest:**

None Declared

